# FNBtools: A Software to Identify Homozygous Lesions in Deletion Mutant Populations

**DOI:** 10.3389/fpls.2018.00976

**Published:** 2018-07-10

**Authors:** Liang Sun, Yinbing Ge, Andrew Charles Bancroft, Xiaofei Cheng, Jiangqi Wen

**Affiliations:** Noble Research Institute, Ardmore, OK, United States

**Keywords:** deletion mutant, homozygous deletion, next generation sequencing, FNBtools, salt tolerance

## Abstract

Deletion mutagenesis such as fast neutron bombardment (FNB) has been widely used for forward and reverse genetics studies in functional genomics. Traditionally, the time-consuming map-based cloning is used to locate causal deletions in deletion mutants. In recent years, comparative genomic hybridization (CGH) has been used to speed up and scale up the lesion identification process in deletion mutants. However, limitations of low accuracy and sensitivity for small deletions in the CGH approach are apparent. With the next generation sequencing (NGS) becoming affordable for most users, NGS-based bioinformatics tools are more appealing. Although several deletion callers are available, these tools are not efficient in detecting small deletions. Population-scale deletion callers that can identify both small and large deletions are rare. We were motivated to create a population-scale deletion detection tool, called FNBtools, to identify homozygous causal deletions in mutant populations by using NGS data. FNBtools is a tool to call deletions at a population-scale and to achieve high accuracy at different levels of coverage. In addition, FNBtools can detect both small and large deletions with the ability to identify unique deletions in a mutant pool by filtering deletions that exist in a wild-type or control pool. Furthermore, FNBtools is also able to visualize all identified deletions in a genome-wide scope by using Circos. From simulated data analysis, FNBtools outperforms four existing popular deletion callers in detecting small deletions at different coverage levels. To test the usefulness of FNBtools in real biological applications, we used it to analyze a salt-tolerant mutant in *Medicago truncatula* and identified the unique deletion locus that is tightly linked with this trait. The causal deletion in the mutant was confirmed by PCR amplification, sequencing and genetic linkage analyses. FNBtools can be used for homozygous deletion identification in any species with reference genome sequences. FNBtools is publicly available at: https://github.com/noble-research-institute/fnbtools.

## Introduction

Induced mutagenesis is a powerful approach in plant breeding and has been widely used for functional genomics. Commonly used mutagens for induced mutagenesis include physical (e.g., fast neutron bombardment [FNB]), chemical (e.g., ethyl methane sulfonate [EMS]) and biological (e.g., T-DNA and transposons) mutagens. For instance, T-DNA has been successfully used to generate large-scale mutant populations in the model plant species *Arabidopsis thaliana* and *Oryza sativa* (Alonso et al., 2003; Sha et al., 2004). In legumes, *Tnt1* and *LORE1* insertion mutagenesis has also been successfully used to tag the *Medicago* *truncatula* and *Lotus* *japonicus* genomes, respectively (d’Erfurth et al., 2003; Malolepszy et al., 2016). Though T-DNA and transposon-based insertion mutagenesis have been widely used for reverse genetics because of their feasibility and convenience in identifying mutated genes (Alonso et al., 2003; Tadege et al., 2008), insertion mutagenesis only creates random mutations in single genes. In many genomes, tandemly repeated genes account for a considerable portion of the genome. These genes are intractable using insertion mutagenesis because their proximity on the chromosomes hinders the creation of higher order mutants. However, deletion mutagenesis achieved by irradiation or FNB can delete adjacent genes (Rogers et al., 2009). Compared to other methods, FNB mutagenesis is easy and effective, and does not require genetic transformation or tissue culture that are typical for T-DNA or *Tnt1* insertion mutagenesis. FNB mutants are non-transgenic and can be grown in fields without regulation.

Fast neutrons are high energy particles that induce a broad range of deletions (from a single base pair to thousands or even millions of base pairs) and other structural variations (SVs) (including combinations of inversions, deletions, substitutions and rearrangements) in cells (Rogers et al., 2009). For example, a deletion of ∼35 kb contains the causal gene *DNF4* that plays an essential role in nitrogen-fixing symbiosis in *M*. *truncatula* (Kim et al., 2015). FNB can also delete small genes in a genome that are relatively hard to tag by insertion mutagenesis (Rogers et al., 2009). In addition, FNB can be used to generate mutant populations with more complete genome coverage than is possible using other approaches (Li et al., 2001). Though comparative genomic hybridization (CGH) and De-TILLING methods identified deletions in FNB mutants (Carter, 2007; Rogers et al., 2009), both methods have low resolution and low accuracy, especially for small deletions. For these reasons, reverse genetic platforms for FNB have not been exploited extensively because of the complexity of mutations generated by FNB. Map-based cloning is a traditional forward genetics methodology to identify causal deletions in FNB mutants. However, this method is time-consuming and expensive. With the rapid development of next generation sequencing (NGS) technologies, whole genome sequencing has become more affordable, providing a new means of identifying causal deletions in FNB mutants.

To identify the causal mutation for a specific FNB mutant phenotype, researchers usually backcross the mutant line to wild-type to obtain a segregating F2 population. If the segregation ratio between wild-type and mutant plants is close to 3:1, this mutant phenotype is likely to be caused by a single recessive mutation. Sequencing pooled mutant DNA and wild-type DNA samples separately is a good strategy for identifying homozygous deletions, which are likely causal deletions.

To call large deletions, mapping-based deletion callers along with paired-end reads are the most commonly used techniques (O’Rourke et al., 2013). The sequence alignment map (SAM) files are successfully used to call small insertions and deletions in bioinformatics tools such as FreeBayes (Garrison and Marth, 2012) and Samtools (Li et al., 2009). Three types of signals from mapping files (e.g., SAM files) can also be used to capture informative reads for large deletions. These signals include: (1) Soft-clipped reads, which occur when one partial fragment of a single read is perfectly mapped to one genomic region and the other partial fragment is perfectly mapped to another nearby genomic region. Pindel (Chiang et al., 2009), Delly (Rausch et al., 2012), and Sprites (Zhang et al., 2016) are all examples of such tools that are currently available to call deletions with soft-clipped read information. (2) Discordant reads, which occur when one read of a pair is mapped to one genomic region and the other is mapped to a different nearby genomic region. Paired-end reads with discordant distance are captured and considered as insertions or deletions. Many tools adopt this signal to call deletions, for example, BreakDancer (Chen et al., 2009), VariationHunter (Hormozdiari et al., 2009; Hormozdiari et al., 2010), and PEMer (Korbel et al., 2009). (3) Read depth-based method, where high-coverage reads are mapped to the up/downstream regions of the deletion site, but fewer or no reads are mapped to the deletion region. Examples of read depth-based methods include SegSeq (Chiang et al., 2009), EWT (Yoon et al., 2009), and CNVnator (Abyzov et al., 2011). However, neither discordant read-based method nor read depth-based method is able to predict the exact positions of breakpoints.

### Challenges of NGS Data in Deletion Analysis

Although several tools are available for deletion calling (Li et al., 2009; McKenna et al., 2010; Koboldt et al., 2012), challenges still exist. These challenges include, but are not limited to, (1) Small and large deletion detection. Small homozygous deletions can cause frame shift and/or introduce early stop codon, leading to disruption of gene function; whereas large deletions cover multiple genes, enabling functional characterization of tandem duplicated genes (Rogers et al., 2009). Despite several calling tools, such as Samtools (Li et al., 2009), GATK (McKenna et al., 2010; DePristo et al., 2011; Van der Auwera et al., 2013), and VarScan (Koboldt et al., 2009, 2012), have been created in the past years. These tools are primarily designed for small deletion callings in human and some animal systems, where naturally occurring small deletions are common but induced large deletions are rare. Therefore, these tools cannot identify homozygous small and large deletions with a high accuracy. Additionally, none of these tools is able to filter out deletions in a control population. (2) Complexity of deletion identification. Given the complexity of identifying deletions, comparing deletions from multiple samples to achieve population-scale studies is still a challenge (Guan and Sung, 2016). (3) Annotation and visualization of deletions. Sometimes a long list of unique homozygous deletions is identified in a mutant sample. To narrow down candidate deletions from the list and focus on potential causal deletions, annotation of the listed candidate deletions facilitates the discovery of causal genes. Overall visualization of candidate deletions across the whole genome can also help generate hypotheses.

### FNBtools as a Solution

To provide a solution to above-mentioned challenges, we employed BWA (Li and Durbin, 2009) and customized PERL and PYTHON scripts to extract and cluster all informative deletion reads from SAM files and created a homozygous deletion-calling tool, FNBtools. FNBtools aims to combine all three types of signals and effectively identify both small and large homozygous deletions in an FNB population. Not only can FNBtools simultaneously analyze multiple samples and identify homozygous deletions, but it can also filter deletions that exist in wild-type or control samples. Users can easily identify unique deletions in mutant samples that are likely to be causal deletions for phenotypes of interest. To better visualize identified deletions, we employed one of the most popular whole genome visualization tools, Circos (Krzywinski et al., 2009). FNBtools integrates the Circos library to generate high-resolution images that can be used for publication.

### A Case Study: Deletion Detection in Salt-Tolerant Mutants in *M. truncatula*

In a joint effort between the Noble Research Institute and the John Innes Centre, an FNB population of 156,000 M2 plants in the *M. truncatula* Jemalong A17 background was generated^1^. In a forward genetic screen for the salt-tolerant phenotype, we isolated two mutants exhibiting enhanced salt tolerance. Backcross and segregation analysis indicated that these mutants are non-allelic, recessive and caused by single locus mutations. To identify the causal locus in each mutant, we sequenced the whole genomes of these mutants using Illumina NextSeq and used FNBtools to test the efficiency of the bioinformatics software.

## Materials and Methods

### Paired-End Mapping

Because paired-end reads are more informative than single-end reads in detecting SV, FNBtools is designed for paired-end reads and is benchmarked by paired-end simulated data. FNBtools aligns all paired-end reads to the reference genome, similar to mapping-based structural variation tools such as Delly, Sprites, and Pindel. To perform alignment, FNBtools uses BWA MEM. BWA MEM produces SAM files, which are used to extract all informative reads.

### Informative Reads Extraction

For small deletions, FNBtools simply extracts informative reads with small deletions from the SAM file produced by BWA MEM. Suppose that a read has a CIGAR string ‘100M5D45M’ in the SAM file where ‘M’ and ‘D’ represent matching and deletion sequences, respectively. The number before the characters represents the number of base pairs involved. This type of small deletion read (5 bp in this example) is extracted and labeled as ‘SMD.’

For large deletions, two signals are used to capture informative reads: discordant reads and soft-clipped reads. Paired-end sequence reads that have discordant distance (i.e., if the inner distance of normal paired-end reads is ∼200 bp, the inner distance of discordant reads might be 1,000 bp) larger than two times the DNA-Seq library fragment length with each pair’s reads perfectly mapped to the reference genome are considered discordant reads. These reads are extracted and labeled as ‘CRR’. For soft-clipped reads, one read is partially aligned to two different genomic positions nearby on the same chromosome. For example, a soft-clipped read has two CIGAR strings ‘mMxS’ and ‘nSyM’ (m and y are the number of base pairs mapped; n and x are the number of soft-clipped base pairs; M indicates matching and S indicates soft-clipping). If the difference between m and n is less than 10 bp, we treat this read as a soft-clipped read, and the read is labeled as ‘CLR’. The SAM information of soft-clipped reads is modified to add deletion bars for large deletions and rewritten to a new SAM file. This new SAM file is sorted and indexed to a binary alignment/map (BAM) file by SAMtools (Li et al., 2009). The deletion bars can be visualized in IGV (Robinson et al., 2011; Thorvaldsdottir et al., 2013) by using the new index BAM file.

### Informative Reads Clustering

All informative reads are represented by a sextuple (seq, chr, st, end, len, type) where seq, chr, st, end, len, and type indicate read id, chromosome, breakpoint position, deletion end position, deletion length, and deletion type (SMD, CLR, or CRR), respectively. They are clustered based on st, end and len. Different types of deletions use different window sizes. For SMD, CLR and CRR deletions, we use a window size of 5 bp, 50 bp and the library mean (500 bp by default), respectively. Informative reads are sorted in ascending order based on the chr and st, and are then clustered into small clusters. In each small cluster, there are three result sets for SMD, CLR and CRR reads separately. For example, three sets for SMD reads are represented by *R_st_smd_*, *R_end_smd_*, and *R_len_smd_*, and similarly for CLR and CRR reads. Because the total number of each set for the same read type should be the same, we used*n_smd_*, *n_clr_*, and *n_clr_* to represent the total number of informative reads for SMD, CLR, and CRR deletions. The preliminary breakpoint position, end position and length of deletions are generated based on clustering of informative reads by using **Table 1**.

**Table 1:** Strategy for determining breakpoint for informative read in one cluster.

Read type	Condition	Breakpoint position *del_st_*	End position *del_end_*	Deletion length *del_len_*
SMD,CLR, CRR	*n_smd_*≥*n_clr_*	*mode(R_st_smd_)*	*mode(R_end_smd_)*	*mode(R_len_smd_)*
SMD,CLR, CRR	*n_smd_ < n_clr_*	*mode(R_st_clr_)*	*mode(R_end_clr_)*	*mode(R_len_clr_)*
CRR	*n_smd_* = 0∩*n_clr_* = 0	*median(R_st_crr_)*	*median(R_end_crr_)*	*median(R_len_crr_)*

*n_smd_, R_st_smd_, R_end_smd_, and R_len_smd_ represent the total number, the breakpoint position, the deletion end position, and deletion length of small deletion reads; n_clr_, R_st_clr_, R_end_clr_, and R_len_clr_ represent the total number, the breakpoint position, the deletion end position, and deletion length of soft clipped reads; n_crr_, R_st_crr_, R_end_crr_, and R_len_crr_ represent the total number, the breakpoint position, the deletion end position, and deletion length of discordant reads; del_st_, del_end_, and del_len_ represent the breakpoint position, end position, and length of preliminary deletions.*

### Gaps in SAM File

If homozygous deletions really exist in a specific genomic region, the read depth of this region should theoretically be zero. This information is very valuable and we used it to filter out heterozygous and false positive deletions. We use BEDTools (Quinlan and Hall, 2010) to capture all gapped regions for each sample by using BAM files (minimal MAPQ 30 to filter out low quality mappings) and compare them with the preliminary deletions from the above clustering step. If the start position of a preliminary deletion is within 3 bp (small deletion) or 20 bp (large deletion with soft clipped reads) or 50 bp (large deletion with only discordant reads) wiggle region of the gap region and the calculative gap length is at least 90% of the preliminary deletion length, we report the deletion as a real homozygous deletion. The detailed criteria are listed in **Table 2**.

**Table 2:** Criteria for homozygous deletion identification.

Read type	Condition
Small deletion (n_*smd*_>0)	n_*smd*_≥ n_*clr*_ ∩ | gap_*st*_ – del_*st*_ | < 3
Large deletion (n_*clp*_ > 0)	n_smd_ < n_clr_ ∩ | gap_st_ – del_set_| < 20 ∩ $\frac{\sum gap_{len}}{del_{len}}$ ≥ 0.9
Large deletion (n_*crr*_ > 0, n_*clp*_ = 0 and n_*smd*_ = 0)	n_clr_ = 0 ∩ n_smd_ = 0 ∩ |gap_st_ – del_st_| < 50 ∩ $\frac{\sum gap_{len}}{del_{len}}$ ≥ 0.9

*n_smd_ and n_clr_ represent the total number of small deletion reads and soft clipped reads respectively. del_st_ and del_len_ represent the breakpoint position and length of preliminary deletions. gap_st_ and ∑gap_len_ represents the start position of gap and the calculative gap length in the deleted region. For large deletion, there is no clipped reads and small deletion reads but only discordant reads, thus the criteria were loosened to allow the difference of gap start position and deletion start position.*

One potential problem in deletion analyses is sample contamination, which can be encountered when multiple samples are pooled. If a pooled sample is contaminated, the causal deletion may not be a homozygous deletion. FNBtools addresses this issue by providing a function to identify all deletions in the mutant pool and calculate deletion frequencies. Users can select those non-homozygous deletions with high frequencies for confirmation purposes if they believe their mutant pool may be contaminated.

The deletion frequency is determined via the following equation:

$$deletion\;frequency=\frac{N_{clr}+N_{smd}+N_{crr}}{N_{clr}+N_{smd}+N_{crr}+N_{der}}*100$$

N_*clr*_ is the number of soft clipped reads spanning deletion region;

N_*smd*_ is the number of small deletions spanning the deletion region;

N_*crr*_ is the number of discordant reads spanning deletion region;

N_*der*_ is the number of reads in the deletion region. Theoretically, N_*der*_ = 0 if deletions are homozygous.

### Unique Deletions Identification

**FIGURE 1:**
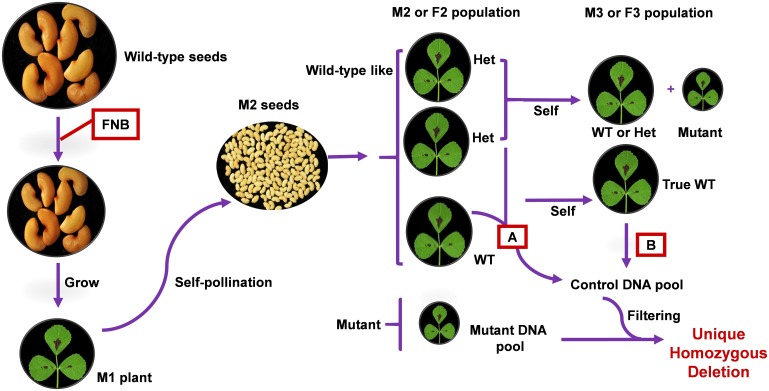
Simplified schema for application of FNBtools to *M. truncatula* FNB population. Once a mutant is isolated from a segregating M2 progeny, it can be directly used as the mutant pool. Alternatively, the mutant can be backcrossed with wild-type to reduce background noise. In this scenario, segregated mutant plant from a F2 progeny can be used as the mutant pool. Pooling strategy A represents the control DNA pool from wild-type and heterozygous individuals in the segregating M2 or F2 progeny. With this control pool, only homozygous deletions commonly existing in the control pool and the mutant pool are filtered out from the mutant pool. Pooling strategy B represents the control DNA pool from true wild-type individuals in the M3 or F3 population. With this control pool, both heterozygous and homozygous deletions commonly existing in the control pool and the mutant pool are filtered out from the mutant pool.

In addition to identifying all homozygous deletions in the mutant population, FNBtools also can filter out deletions in wild-type (control) samples. FNBtools provides two options to filter out deletions from the control pool depending on the composition of the control pool. (1) Filter out homozygous deletions commonly existing in the control (wild-type) pool (see **Figure 1** for the pooling strategy A). In this case, the control pool contains both real wild-type individuals and heterozygous individuals from a segregating M2 or F2 progeny; therefore, only homozygous deletions that commonly exist in the mutant pool and the control pool are filtered out. (2) Filter out homozygous and heterozygous deletions commonly existing in the control pool (see **Figure 1** for pooling strategy B). In this case, only real wild-type individuals (no segregation of mutants is observed from selfed M2 or F2 wild-type-like individuals) in M3 or F3 generation are pooled as the control pool. Since the control pool is pure wild-type, there are no heterozygous deletions for the causal locus, both homozygous and heterozygous deletions commonly existing in the control pool and the mutant pool are filtered out. Only deletions that uniquely exist in the mutant population are reported by FNBtools. If users prefer to include all homozygous deletions regardless of uniqueness, FNBtools accepts a parameter to toggle what is reported in this regard. FNBtools currently uses fixed cut-off values for supporting reads > = 3 to reliably identify homozygous deletions.

### Annotation and Visualization of Deletions

If deletions fall in gene regions (including 5′UTR and 3′UTR), exons and introns are annotated by gene IDs. These deletions and associated gene IDs can be visualized in Circos by FNBtools. In the Circos graph, there are three layers of visualization. The outermost layer shows deletion lengths smaller than 100 bp. The middle layer shows deletions with a length between 100 bp and 1 kb. The innermost layer shows deletion lengths greater than 1 kb.

### Plant Materials and Sequencing

Wild-type *M. truncatula* (ecotype Jemalong A17) seeds were mutagenized by fast neutron irradiation at the 35 Gy dosage level. Approximately 8,600 M2 plants derived from 1,720 M1 lines were screened on half-strength Murashige-Skoog (1/2 MS) medium containing 1.3% NaCl and 0.5% Phytogel, resulting in the isolation of two salt-tolerant mutants, S1 and S2. To generate sequencing materials, we separately backcrossed the two mutants to wild-type A17. Resultant BC1F1 plants were self-pollinated and the seeds produced were grown to produce BC1F2 plants. Segregating F2 seeds were scarified with concentrated sulfuric acid for 8 min and washed thoroughly with tap water. The scarified seeds were further sterilized with 30% bleach for 10 min, followed by extensive rinsing with autoclaved ddH_2_O and cold treatment at 4°C for 7 days on solidified 1/2 MS medium. Germinated seeds were first grown on regular 1/2 MS for 7 days in a growth chamber with a regime of 18 h light/25°C and 6 h dark/22°C photoperiod. At least 50 1-week-old F2 seedlings from each mutant were transferred onto solidified 1/2 MS medium containing 1.3% NaCl for 2 weeks. Surviving plants are salt-tolerant and considered mutants, while dead plants are wild-type or heterozygous. Because wild-type and heterozygous plants from the segregating progenies were dead during the salt selection, no materials were left to be the control. In this case, we used the mutant S1, which has the same parental background as mutant S2 but is not allelic to S2, as the control for FNBtools data processing and analysis. One trifoliate leaf from each surviving seedling was collected and frozen in liquid nitrogen for DNA isolation. Genomic DNA from 10 mutant S2 seedlings and 10 control S1 seedlings were isolated individually using the Dellaporta miniprep method (Weigel and Glazebrook, 2009) and pooled as the mutant and the control DNA samples for sequencing. The integrity of each DNA sample was visually examined on a 1% agarose gel. DNA concentration and purity were assessed using an Agilent 2100 Bioanalyzer (Agilent, Santa Clara, CA, United States). DNA samples were submitted to the Genomics Core Facility at Noble Research Institute for 150 bp paired-end sequencing on an Illumina NextSeq 500 sequencing system. From the same segregating F2 progeny, 65 S2 mutant plants were sampled for genomic DNA isolation and subsequent genetic linkage analysis.

## Results and Discussion

### Experimental Design

Our deletion mutant population design derives from MutMap (Abe et al., 2012). The principle of FNBtools is illustrated using FNB mutants in *M. truncatula* (**Figure 1**) because we have a large FNB mutant collection in this legume species. The approach is designed for mutagenized plants (M0) that have been used to generate M1 mutant lines, which have been selfed to generate an M2 population. Multiple mutant plants identified from a segregating M2 progeny can be directly used as the mutant pool. Alternatively, the mutant can be backcrossed with a wild-type plant to reduce background noise. Several mutant plants are then selected from the F2 progeny and pooled to be used as the mutant pool. This has the advantage of averaging-out non-causal mutations. To better pinpoint the causal homozygous deletions from a long list of identified deletions in a mutant pool (sometime the deletions can number in the thousands), a wild-type or control pool is used. We used two strategies for control sample pooling (**Figure 1**). In pooling strategy A, the control pool consists of individuals from a segregating M2 or F2 progeny that have a wild-type phenotype (containing a mixture of individuals that are homozygous wild-type and heterozygous mutant). In pooling strategy B, only true wild-type individuals, identified by progeny testing of the M3 or F3 generation, are pooled as the control. The pooled mutant and control DNA samples are sequenced separately using Illumina Hiseq or NextSeq. With the control pool from strategy A, only homozygous deletions commonly existing in the control pool and the mutant pool are filtered out from the mutant pool. With the control pool from strategy B, both heterozygous and homozygous deletions commonly existing in the control pool and the mutant pool are filtered out from the mutant pool.

**FIGURE 2:**
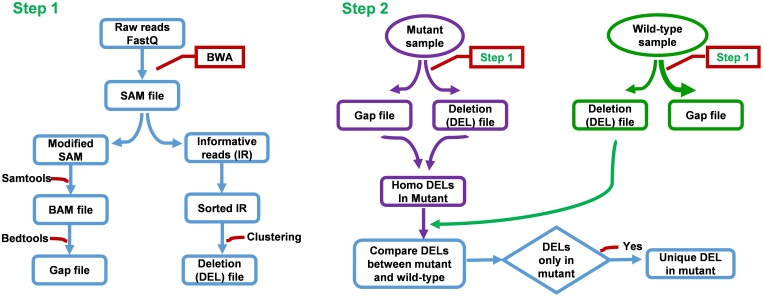
Flow chart of FNBtools. Step 1 aligns NGS reads to the reference genome. Step 2 compares the deletions between the mutant sample and the wild-type or the control sample.

FNBtools is able to accept multiple samples and align all reads to the reference genome. **Figure 2** provides additional details about our FNBtools in the Materials and Methods section. A flowchart summarizing the methodology is shown in Supplementary Figure S2.

### Simulation Data

FNBtools was benchmarked on simulated data. The *M. truncatula* A17 genome (version 4.0 ∼400 Mb) (Tang et al., 2014) was used to generate random deletions using SVsim.^2^ A total of 315 deletions with different deletion sizes were generated. The distribution of deletion sizes is shown in Supplementary Figure S1. Based on the mutated *M. truncatula* genome, 150 bp paired-end reads with 5x, 10x, 20x, and 40x coverage were generated using wgsim (Li et al., 2009), assuming a 0.5% sequencing error rate under each deletion size. To test the functionality of filtering heterozygous deletions in the wild-type sample, we also generated heterozygous 150 bp paired-end reads for the wild-type sample with 0.05, 0.1, 0.2, and 0.5 deletion frequencies at 5x, 10x, 20x, and 40x coverage, respectively.

### Comparison With Similar Tools

We chose deletion callers such as Pindel, BreakDancer, Delly, and Sprites to compare with FNBtools. For a large deletion (i.e., *n_smd_* = 0, *n_smd_* represents the total number of small deletion reads) be counted as a successful detection, the breakpoints of deletion positions should be ±100 bp from the breakpoints of true deletions, and the deletion length should be 90% overlapping with the true deletions in simulated data. For small deletions (*n_smd_* > 0), the breakpoints of each deletion position should be ±5 bp from the breakpoints of true deletions, and the overlapping rate should be 50%. Recall and precision values were measured together with calculating an accuracy score, F-score, described below:

$$\text{Precision}=\frac{TP}{TP+FP}\text{TP: true positive; FP: false positive}$$

$$\text{Recall}=\frac{TP}{TP+FN}\text{TP: true positive; FN: false negative}$$

F-score=2*precision*Recallprecision+Recall

**FIGURE 3:**
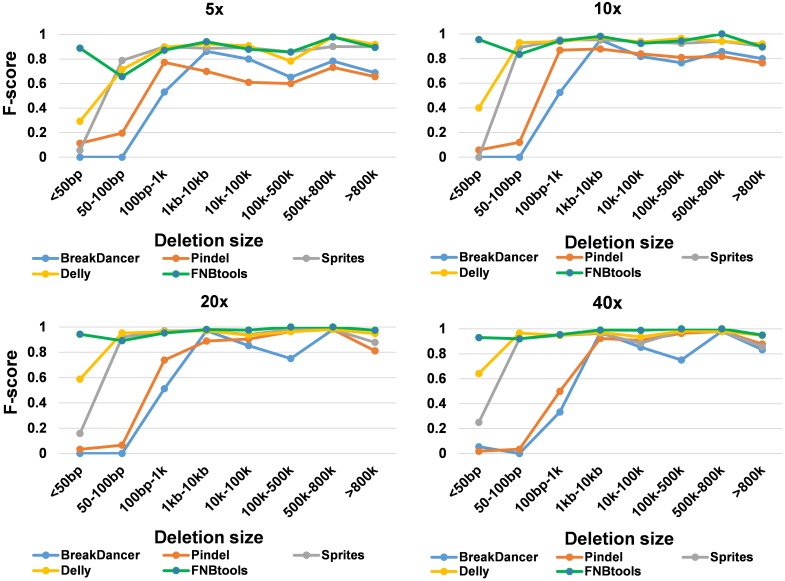
Accuracy summary of five deletion callers with different coverage simulated data.

**FIGURE 4:**
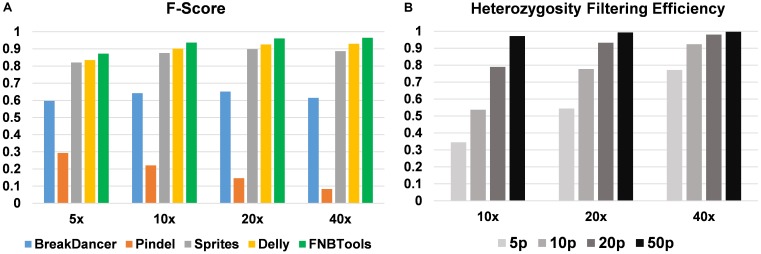
**(A)** Performance comparison of homozygous deletion detection. **(B)** Heterozygosity filtering efficiency by FNBtools. 5p, 10p, 20p, and 50p represent 5, 10, 20, and 50% deletion frequencies in the wild-type sample.

To evaluate how FNBtools, Pindel, BreakDancer, Delly, and Sprites perform at different deletion sizes, we visualized the performance of all five tools for comparison in **Figures 3**, **4A**. In terms of *F*-score, FNBtools outperforms almost all other tools for detecting homozygous deletions at different coverage levels. We found that FNBtools has a high *F*-score at almost every deletion size range, indicating that FNBtools performs very well even at low coverage. It is noteworthy that FNBtools has high precision and recall values at small deletions, which commonly occur in FNB populations. In contrast, the other four tools have a limited ability to detect small deletions. Sequencing at lower coverage can greatly reduce the cost of NGS. Furthermore, from a comparison analysis using masked and unmasked *Medicago truncatula* reference genome, we found that FNBtools can also detect deletions in repetitive regions (Supplementary Figure S3).

FNBtools includes a function to filter deletions that exist in the wild-type (or control) population to identify unique deletions in the mutant population (see Materials and Methods for details). To validate the efficiency of this filtering function, we compared the results at different coverage levels and deletion frequencies. We found that the filtering efficiency increases as either the coverage level or deletion frequency increases. When the deletion frequency is 0.5 (50%) in the wild-type population, 97.2, 99.3, and 99.7% non-unique deletions can be filtered out at 10x, 20x, and 40x coverage levels, respectively (see **Figure 4B**).

### Real Biological Data

**FIGURE 5:**
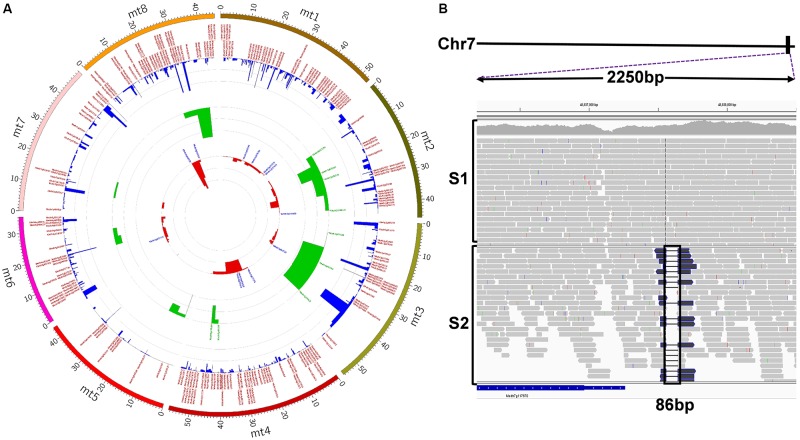
**(A)** Circos visualization of identified deletions in S2. The inner-most circle (red) represents deletions > 1kb; the middle circle (green) represents deletions between 100 bp and 1 kb; the out-most circle (blue) represents deletions < = 100 bp. Each circle was annotated by gene IDs if deletions contain or are in genes (high-resolution image link: https://github.com/noble-research-institute/fnbtools/blob/master/example/Circos_S2_deletion.png). **(B)** Visualization of the causal deletion using IGV. The deletion is located at the bottom of chromosome 7 (solid black rectangular box). A 2250 base pair window from IGV, as indicated by the purple dotted lines, shows the reads around the deletion. The histogram plot illustrates the read depth with individual reads plotted below. There is no deletion in the control sample S1. Black box highlights the 86bp deletion region in the mutant sample S2.

To further evaluate the usefulness of FNBtools in real biological samples, we employed the tool in the identification of the causal deletion in a salt-tolerant FNB mutant. In most cases, wild-type (strategy B) or wild-type-like plants (strategy A) from a segregating progeny (**Figure 1**) are pooled and used as the control. However, in our case study, since wild-type and heterozygous plants from the segregating progenies were dead during the salt selection, no materials were left to be used as the control. In this case, we used pooled DNA from another mutant S1, which has the same parental background as the S2 mutant but is not allelic to S2, as the control for FNBtools data processing and analysis (see Materials and Methods for more details on sample pooling). The S1 and S2 pools were then sequenced using the Illumina NextSeq platform to generate paired-end reads. Approximately 131 million and 45 million raw reads from samples S1 and S2, respectively, were obtained from sequencing. After filtering out low quality reads, ∼112 million and 39 million clean reads for S1 and S2, respectively, were used for analysis. The coverage of the control and the mutant sample is 93x and 33x, respectively. These clean reads were mapped to the *M. truncatula* reference genome, and homozygous deletions were called using FNBtools. In total, 28,637 deletions were identified in sample S2 (Supplementary File S1). Out of these deletions, 5,373 are homozygous deletions that can be visualized in **Figure 5A** (high-resolution image link^3^). We also identified 2651 homozygous deletions in the control sample S1. Interestingly, we found 1,542 homozygous deletions that are present at the exact same locations with exact same deletion sizes in both control (S1) and mutant (S2) samples, indicating these deletions are systematically present in our materials. One explanation for this observation is that the starting materials we used for mutagenesis are different from the materials used for reference genome sequencing.

To confirm the accuracy of FNBtools in determining homozygous deletions, we randomly selected 23 deletions with different read coverages for examination in the S1 sample. Supplementary Table S1 shows that when a deletion has four or more informative reads (either SMD or CLR), the deletion prediction by FNBtools is accurate with a 100% success rate. When a deletion has three reads, the prediction has a 74% accuracy. When the coverage of NGS data is very high, for example 93x coverage in our case study, we recommend to use more stringent cut-off values for supporting reads number.

From reciprocal genetic crosses, we knew that S1 and S2 are non-allelic mutant lines and the phenotype is caused by different causal genes/deletions. We filtered out all homozygous deletions from S2 that are either heterozygous or homozygous in S1. After filtering out deletions that commonly exist in S1 and S2, 12 unique homozygous deletions were identified in S2. All of these unique deletions have at least 12 informative reads, and the deletion sizes and positions were confirmed by PCR amplification and sequencing.

To find the causal deletion(s) in the mutant, we performed a genetic linkage analysis for these 12 deletions using a small population consisting of 65 mutant samples segregated from a backcrossed F2 progeny. Theoretically, based on traditional parametric linkage analysis, if a homozygous deletion is present in 25% of mutant samples, this deletion is recombining freely during meiosis and is not linked with the causal mutation (Morton, 1955). This number may fluctuate depending on the population size and the mutation location on chromosomes. If a deletion is the causal mutation, it should be present in all mutants. As shown in Supplementary Table S2, deletion 11 is present in all mutant plants, indicating strong linkage with the phenotype. All other deletions show free segregation patterns; thus, they are not linked with the phenotype. However, deletion 11 falls in the intergenic region between Medtr7g117670 and Medtr7g117675 (see **Figure 5B**). Though we successfully identified the linked locus, it will take more effort to pinpoint the causal gene. One approach is to identify mutants of these two genes from the *M. truncatula Tnt1* insertion mutant population (Tadege et al., 2008) and examine whether the mutants show a salt tolerance phenotype.

## Conclusion

We have developed a software, FNBtools, which can identify both small and large homozygous deletions in FNB populations. FNBtools was developed by taking two types of reads (soft-clipped reads and discordant reads) into consideration. Using simulated data, FNBtools outperforms existing popular deletion callers, BreakDancer, Pindel, Delly, and Sprites, in detecting small deletions in all tested coverage levels. In a real biological case study using FNBtools, we successfully identified a locus linked with a phenotype in an FNB mutant from a *M. truncatula* mutant population. The linkage between the identified causal deletion and exhibited phenotype in the mutant was confirmed by PCR in a small segregating population. In plants, many deletion mutant populations have been generated by different groups, for example, *Arabidopsis thaliana* (Belfield et al., 2012), soybean (Bolon et al., 2011), peanut (Wang et al., 2015), common bean (O’Rourke et al., 2013), etc. Furthermore, the genome sequences of these plant species are also available. We speculate that FNBtools can be used for deletion detection in these mutant populations. With the ease of NGS sequencing, more plant species have been and will be sequenced. In fact, as long as genome sequences are available, FNBtools can be used for deletion identification in any species, such as microbes, worms and fruit flies. Application of FNBtools in crops will provide a quick and reliable tool in non-transgenic molecular breeding.

Our FNBtools is currently a reference genome-based tool. If deletions occur in the gap regions of the reference genome, for example, an entire scaffold is deleted, it is hard to identify this type of deletion.

## Availability

FNBtools is an open source program available in the GitHub repository (https://github.com/noble-research-institute/fnbtools).

## Author Contributions

LS and JW conceived the original research plans. LS, YG, and AB performed all bioinformatics analysis. XC performed most of the experiments. LS and JW supervised and complemented the writing.

## Conflict of Interest Statement

The authors declare that the research was conducted in the absence of any commercial or financial relationships that could be construed as a potential conflict of interest.

## Footnotes

1 https://medicago-mutant.noble.org/mutant/index.php

2 https://github.com/GregoryFaust/SVsim

3 https://github.com/noble-research-institute/fnbtools/blob/master/example/Circos_S2_deletion.png
