# Supplementary material for: FNBtools: A Software to Identify Homozygous Lesions in Deletion Mutant Populations
Source: Front Plant Sci. 2018 Jul 10;9:976. doi: 10.3389/fpls.2018.00976 (PMC6048286; doi:10.3389/fpls.2018.00976)
Supplement: Supplementary file 1 [file Presentation_1.PDF]

# FNBtools: A Software to Identify Homozygous Deletions in FNB Mutant Populations

## Supplemental Material

**Files in this Data Supplement:**

- **Supplementary_Table S1.pdf**
- **Supplementary_Table S2.pdf**
- **Supplementary_Fig_S1.pdf**
- **Supplementary_Fig_S2.pdf**
- **Supplementary_Fig_S3.pdf**
- **Supplementary_file1.excel**

## Supplemental Table S1: Accuracy test for sample S1

| Deletion ID | Chr | Breakpoint Start | Breakpoint End | Deletion Size (bp) | Supportive Read Number | Gap Starts | Gap Ends | Del_mutant | Del_control | Homo_Unique | Confirmation |
|---|---|---|---|---|---|---|---|---|---|---|---|
| 25 | chr1 | 1284048 | 1284049 | 1 | CLR=0;CRR=0;SMD=3;FLR=2;FRR=26 | 1284048 | 1284049 | Yes | Yes | No | No |
| 109 | chr1 | 7702199 | 7702205 | 6 | CLR=0;CRR=0;SMD=3;FLR=7;FRR=3 | 7702199 | 7702205 | Yes | Yes | No | Yes |
| 267 | chr1 | 29717335 | 29717336 | 1 | CLR=0;CRR=0;SMD=3;FLR=3;FRR=3 | 29717335 | 29717336 | Yes | Yes | No | Yes |
| 540 | chr2 | 15321685 | 15321687 | 2 | CLR=0;CRR=0;SMD=3;FLR=6;FRR=2 | 15321685 | 15321687 | Yes | Yes | No | Yes |
| 543 | chr2 | 15331444 | 15331445 | 1 | CLR=0;CRR=0;SMD=3;FLR=3;FRR=3 | 15331444 | 15331445 | Yes | No | Yes | Yes |
| 755 | chr3 | 21965675 | 21965676 | 1 | CLR=0;CRR=0;SMD=3;FLR=3;FRR=8 | 21965675 | 21965676 | Yes | Yes | No | Yes |
| 768 | chr3 | 23588052 | 23588053 | 1 | CLR=0;CRR=0;SMD=3;FLR=3;FRR=2 | 23588052 | 23588053 | Yes | No | Yes | Yes |
| 985 | chr4 | 13164475 | 13164479 | 4 | CLR=0;CRR=0;SMD=3;FLR=12;FRR=3 | 13164475 | 13164479 | Yes | Yes | No | No |
| 275 | chr1 | 31035828 | 31035847 | 19 | CLR=3;CRR=0;SMD=0;FLR=3;FRR=4 | 31035828 | 31035847 | Yes | Yes | No | No |
| 709 | chr3 | 12721653 | 12721704 | 51 | CLR=3;CRR=0;SMD=0;FLR=7;FRR=5 | 12721654 | 12721704 | Yes | Yes | No | Yes |
| 958 | chr4 | 11234103 | 11234221 | 118 | CLR=3;CRR=0;SMD=0;FLR=4;FRR=4 | 11234103 | 11234221 | Yes | Yes | No | No |
| 1279 | chr4 | 43194196 | 43194314 | 118 | CLR=3;CRR=0;SMD=0;FLR=16;FRR=18 | 43194197 | 43194313 | Yes | Yes | No | No |
| 1620 | chr6 | 4956772 | 4956804 | 32 | CLR=3;CRR=0;SMD=0;FLR=3;FRR=11 | 4956772 | 4956804 | Yes | Yes | No | Yes |
| 12 | chr3 | 23588052 | 23588053 | 1 | CLR=0;CRR=0;SMD=3;FLR=3;FRR=2 | 23588052 | 23588053 | Yes | No | Yes | Yes |
| 24 | chr5 | 30952197 | 30952198 | 1 | CLR=0;CRR=0;SMD=3;FLR=3;FRR=4 | 30952197 | 30952198 | Yes | No | Yes | No |
| 38 | chr8 | 42384569 | 42384573 | 4 | CLR=0;CRR=0;SMD=3;FLR=2;FRR=3 | 42384569 | 42384573 | Yes | No | Yes | Yes |
| 666 | chr3 | 3928202 | 3928277 | 75 | CLR=4;CRR=0;SMD=0;FLR=4;FRR=4 | 3928202 | 3928277 | Yes | Yes | No | Yes |
| 700 | chr3 | 10421730 | 10421774 | 44 | CLR=4;CRR=0;SMD=0;FLR=4;FRR=13 | 10421730 | 10421774 | Yes | Yes | No | Yes |
| 1 | chr1 | 3325600 | 3325601 | 1 | CLR=0;CRR=0;SMD=4;FLR=5;FRR=5 | 3325600 | 3325601 | Yes | No | Yes | Yes |
| 25 | chr5 | 33844610 | 33844611 | 1 | CLR=0;CRR=0;SMD=4;FLR=3;FRR=6 | 33844610 | 33844611 | Yes | No | Yes | Yes |
| 42 | scaffold0124 | 26758 | 26759 | 1 | CLR=0;CRR=0;SMD=4;FLR=4;FRR=2 | 26758 | 26759 | Yes | No | Yes | Yes |
| 27 | chr6 | 30824164 | 30824165 | 1 | CLR=0;CRR=0;SMD=6;FLR=6;FRR=6 | 30824164 | 30824165 | Yes | No | Yes | Yes |
| 10 | chr2 | 26959662 | 26959663 | 1 | CLR=0;CRR=0;SMD=7;FLR=6;FRR=6 | 26959662 | 26959663 | Yes | No | Yes | Yes |

## Supplemental Table S2: Genetic linkage analysis of 12 unique homozygous deletions in sample S2

| Deletion ID | Chr | Start_position | End_position | Deletion size (bp) | Supportive reads | Gap Starts | Gap Ends | Del_mutant | Del_control | Homo_Unique | Annotation | Linkage |
|---|---|---|---|---|---|---|---|---|---|---|---|---|
| 1 | chr1 | 42780901 | 42780902 | 1 | CLR=0;CRR=0;SMD=20;FLR=20;FRR=18 | 42780901 | 42780902 | Yes | No | Yes | ['Medtr1g095050'] | 25% |
| 2 | chr2 | 4912716 | 4912717 | 1 | CLR=0;CRR=0;SMD=8;FLR=9;FRR=8 | 4912716 | 4912717 | Yes | No | Yes | ['Medtr2g016200'] | 31% |
| 3 | chr2 | 29170197 | 29170198 | 1 | CLR=0;CRR=0;SMD=9;FLR=8;FRR=9 | 29170197 | 29170198 | Yes | No | Yes | [] | 43% |
| 4 | chr3 | 34323306 | 34323314 | 8 | CLR=0;CRR=0;SMD=19;FLR=20;FRR=19 | 34323306 | 34323314 | Yes | No | Yes | ['Medtr3g075410'] | 35% |
| 5 | chr4 | 28247742 | 28247749 | 7 | CLR=0;CRR=0;SMD=13;FLR=14;FRR=14 | 28247745 | 28247749 | Yes | No | Yes | ['Medtr4g074220'] | 23% |
| 6 | chr4 | 52426842 | 52426846 | 4 | CLR=0;CRR=0;SMD=23;FLR=25;FRR=24 | 52426842 | 52426846 | Yes | No | Yes | ['Medtr4g126140'] | 41% |
| 7 | chr5 | 2830713 | 2830715 | 2 | CLR=0;CRR=0;SMD=24;FLR=25;FRR=24 | 2830713 | 2830714 | Yes | No | Yes | ['Medtr5g010520'] | 42% |
| 8 | chr5 | 17668358 | 17668360 | 2 | CLR=0;CRR=0;SMD=16;FLR=15;FRR=15 | 17668358 | 17668360 | Yes | No | Yes | [] | 37% |
| 9 | chr5 | 41319722 | 41319732 | 10 | CLR=0;CRR=0;SMD=14;FLR=19;FRR=15 | 41319722 | 41319732 | Yes | No | Yes | [] | 32% |
| 10 | chr7 | 28316601 | 28316609 | 8 | CLR=0;CRR=0;SMD=20;FLR=23;FRR=26 | 28316603 | 28316609 | Yes | No | Yes | ['Medtr7g075260'] | 35% |
| 11 | chr7 | 48837558 | 48837644 | 86 | CLR=19;CRR=0;SMD=0;FLR=22;FRR=25 | 48837558 | 48837643 | Yes | No | Yes | [] | 100% |
| 12 | scaffold0352 | 2591 | 2597 | 6 | CLR=0;CRR=0;SMD=18;FLR=20;FRR=18 | 2591 | 2597 | Yes | No | Yes | [] | 68% |

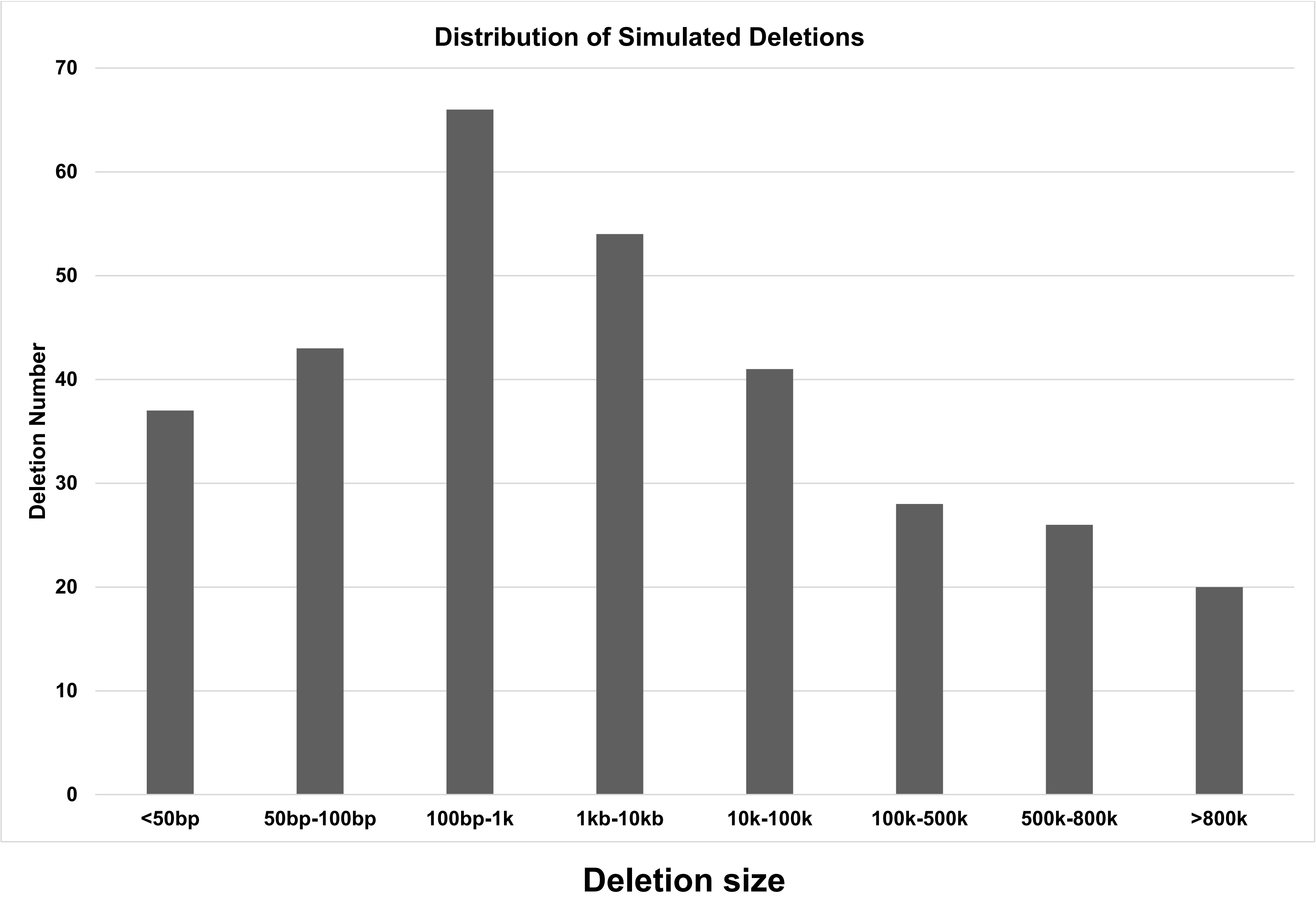


**Supplemental Figure S1. Distribution of simulated deletions in *Medicago truncatula* genome A17**

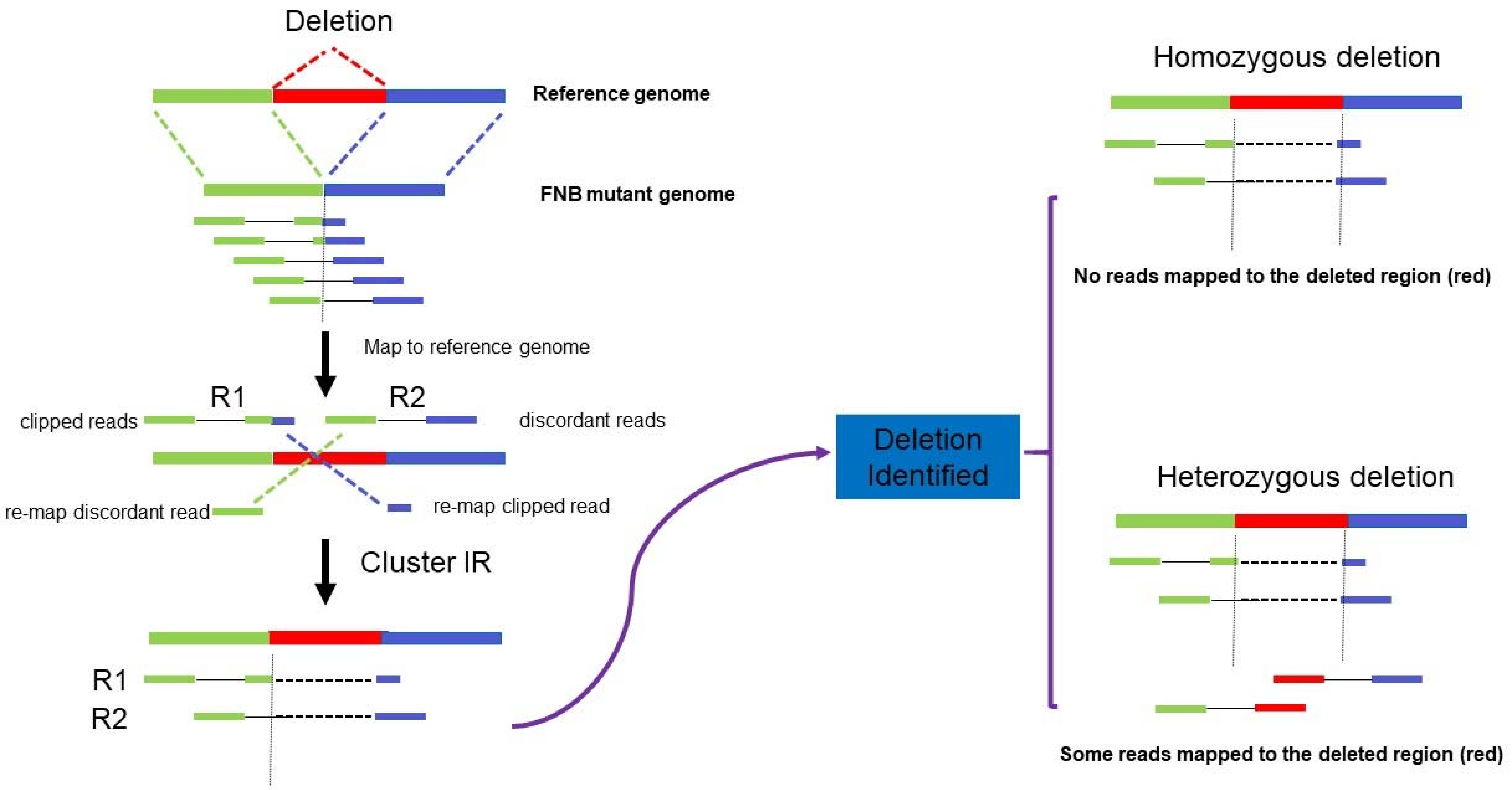


**Supplemental Figure S2. A methodology flowchart for deletion detection. Step 1: map all reads (R1, R2, and so on) to the reference genome and extract all informative reads (IR) from SAM files. Step 2: cluster all informative reads based on mapping positions. Step 3: identify homozygous deletions according to the gap information**

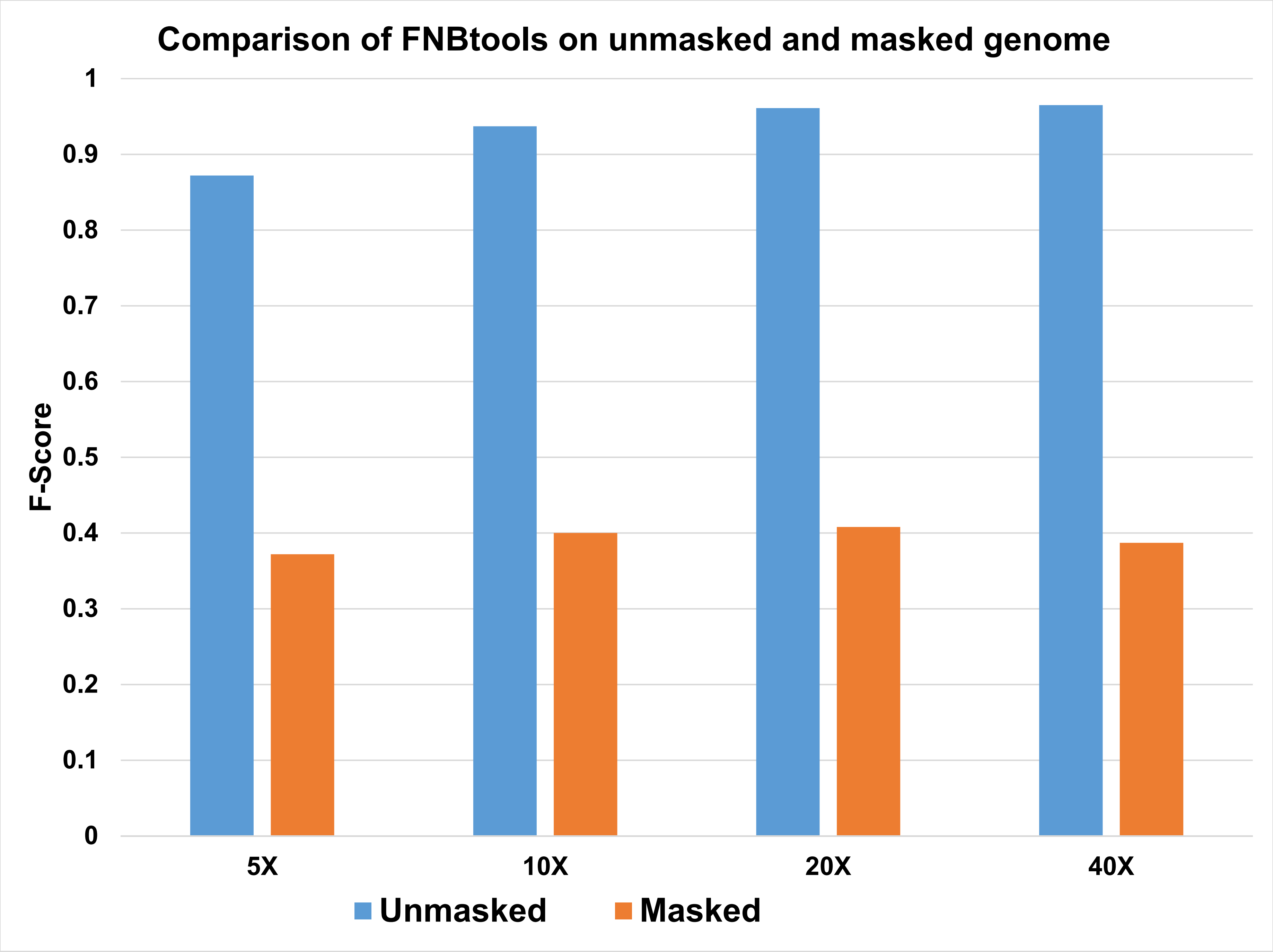


**Supplemental Figure S3. Comparison of FNBtools performance in unmasked and masked *Medicago truncatula* genome. Significantly reduced F-scores were observed from the masked reference genome due to undetectable deletions in the masked repetitive regions.**
